# Coumarin Antifungal Lead Compounds from *Millettia thonningii* and Their Predicted Mechanism of Action

**DOI:** 10.3390/molecules21101369

**Published:** 2016-10-15

**Authors:** Daniel M. Ayine-Tora, Robert Kingsford-Adaboh, William A. Asomaning, Jerry J.E.K. Harrison, Felix C. Mills-Robertson, Yahaya Bukari, Patrick O. Sakyi, Sylvester Kaminta, Jóhannes Reynisson

**Affiliations:** 1 School of Chemical Sciences, University of Auckland, 23 Symonds Street, 1142 Auckland, New Zealand; dayi479@aucklanduni.ac.nz; 2 Department of Chemistry, University of Ghana, LG 56, Legon-Accra, Ghana; kadabohs@yahoo.com (R.K.-A.); waasoman@ug.edu.gh (W.A.A.); harriebow@gmail.com (J.J.E.K.H.); poskeydinho@yahoo.com (P.O.S.); 3 Department of Biochemistry and Biotechnology, Kwame Nkrumah University of Science and Technology, Kumasi, Ghana; mirobfc2002@yahoo.com; 4 Botany Department, University of Ghana, LG 55, Legon-Accra, Ghana; sparrowson@yahoo.com; 5 Centre for Plant Medicine Research, 73, Mampong-Akuapem, Ghana; slykmt@yahoo.co.uk

**Keywords:** natural products, *Candida albicans*, molecular modelling, CYP51, *Sclorotium*, isoflavone

## Abstract

Fungal pathogens continue to pose challenges to humans and plants despite efforts to control them. Two coumarins, robustic acid and thonningine-C isolated from *Millettia thonningii*, show promising activity against the fungus *Candida albicans* with minimum fungicidal concentration of 1.0 and 0.5 mg/mL, respectively. Molecular modelling against the putative bio-molecular target, lanosterol 14α-demethylase (CYP51), revealed a plausible binding mode for the active compounds, in which the hydroxyl group binds with a methionine backbone carboxylic group blocking access to the iron catalytic site. This binding disrupts the synthesis of several important sterols for the survival of fungi.

## 1. Introduction

*Candida albicans* is one of the most common fungal pathogens causing infection despite major efforts to control it [1,2]. *C. albicans* has emerged as one of the main causes of morbidity and mortality in immunocompromised patients suffering from diseases such as cancer or AIDS [3,4,5,6]. Candidiasis infection can affect the skin, oral cavity, oesophagus, gastrointestinal tract, vagina and vascular system of humans [7,8,9]. There are a number of drugs such as flucoconazole, nystatin, voriconazole, terbinafine and echinocandin to address fungal infections [10]. However, drug resistance, restricted systemic usage due to dose-related toxicity and the emergence of new strains of fungal infections all undermine the efficacy of these drugs [11,12,13,14,15]. Hence, it is necessary to develop new antifungal treatments to address these emerging challenges.

*Milletia thonningii* (Schum-Thonn) Baker is a deciduous tree that grows in tropical climates. It belongs to the family Papilionaceae and is indigenous to West and Central Africa [16,17,18]. Preparations from this tree are used in traditional medicine for alleviating several diseases and disorders such as dysentery, intestinal pain, worms, tuberculoid leprosy, menstrual disorder, measles and chicken pox [16,18]. In addition, the seeds show molluscicide activity [19] and the juice from the leaves is lethal to *Bulinus* snails, the host for schistosomiasis [20].

The many uses of *M. thonningii* as a traditional source of remedies have attracted research into its constituents. For instance, some isoflavones isolated from this plant were reported to demonstrate lethal toxicity to brine shrimp [21] as well as to inhibit activation of hypoxia-inducible factor-1 (HIF-1) in human breast tumour T47D cells [22]. Interestingly, coumarin derivatives have been shown to demonstrate antifungal activity [23,24,25,26].

The aim of this work was to investigate the activity of the constituent of *M. thonningii* against *C. albicans* and investigate a mechanism against lanosterol 14α-demethylase (CYP51), a common anti-fungal target. This enzyme converts lanosterol to ergosterol, the disruption of this enzyme results in structural changes of plasma membranes, and causes a change in the concentrations of various 14α-methyl sterols, which has cytostatic and cytotoxic consequences [27,28,29]. It is hoped that the findings will provide new lead compounds as well as useful insights into the rational design of the active compounds of *M. thonningii* for antifungal treatment.

## 2. Results and Discussion

### 2.1. Isolation and Synthesis

**Figure 1:**
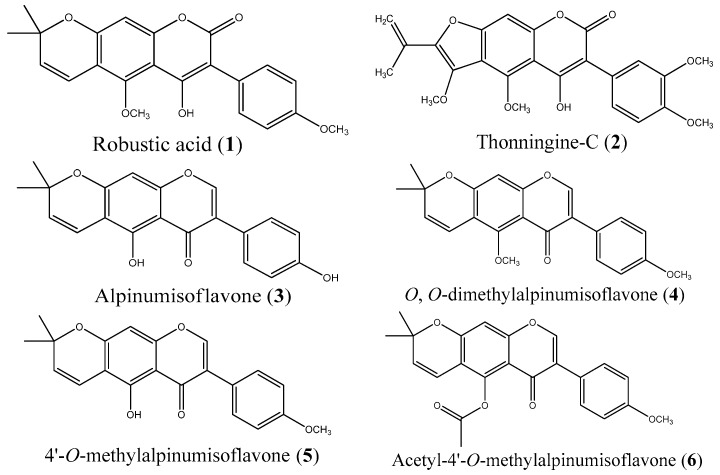
Structures of isolated coumarins (**1**, **2**) and isoflavanones (**3**, **4**, **5**) from *M. thonningii* and a synthesised derivative (**6**).

In order to investigate the activity of the compounds from *M. thonningii* against *C. albicans*, the constituents of the seeds of *M. thonningii* were isolated. The isolation yielded robustic acid (**1**), thonningine-C (**2**), alpinumisoflavone (**3**), *O*,*O*-dimethylalpinumisoflavone (**4**) and 4’-*O*-methylalpinumisoflavone (**5**). These compounds are known constituents of the plant [20,30,31,32]. The yield of compound **5** was low but more was successfully synthesised via demethylation of **4** for biological testing. Compound **5** was also acetylated to form a new derivative, acetyl-4-*O*-methylalpinumisoflavone (**6**), to help understand the structure activity relationships of the chemical series. The molecular structures are displayed in Figure 1.

### 2.2. Investigation into the Selective Demethylation of Isoflavone ***4***

Previous studies focused on determination of reagents to give optimum yields for demethylation of compounds such as visnagen and khellin. According to these investigations, a combination of formic acid and KI gives the optimal yield. Replacing KI with NaBr, KBr, and NaI did not improve the yield of this reaction. In addition, using glacial acetic acid instead of formic acid resulted in a lower yield. Therefore, formic acid and KI were used for the demethylation of isoflavone **4** to synthesise derivative **5** [33].

**Figure 2:**
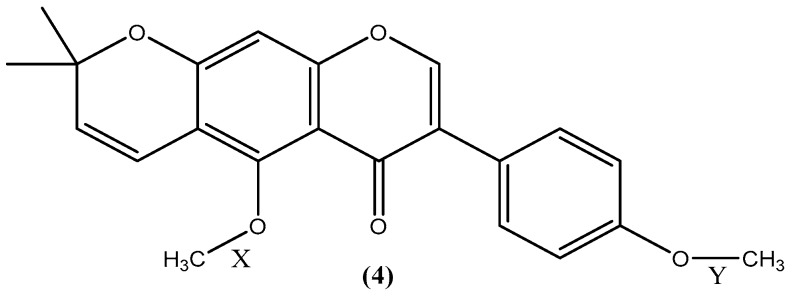
Structure of *O*,*O*-dimethylalpinumisoflavone (**4**) and the two carbon-oxygen bonds under investigation X and Y.

Interestingly, the demethylation of isoflavone **4** only occurred at the methoxy group on the benzopyrone moiety (X) but not on the phenyl group (Y) (see Figure 2). This is in line with a previous investigation, which showed that methoxy groups *ortho* to acyl moieties are highly reactive and acid sensitive compared to aromatic methoxy groups [34]. In order to shed light on this phenomenon, thermochemical density functional theory (DFT) calculations were performed to determine bond dissociation energies (BDE) for the bonds X and Y, and proton affinities (PA) for derivative **4**. It has been shown that BDEs and PAs can be reliably calculated using the DFT method [35,36,37,38]. PAs of the carbonyl and methoxy oxygen atoms were calculated to determine the preferred protonation site due to the acidic nature of the reaction medium. The PA values show that the carbonyl oxygen is the preferred protonation site (see Table S1 in the Supplemetary Information). The BDEs of neutral and protonated forms of **4** were determined for both homolytic and heterolytic cleavages of the O-C bond and the results are shown in Table 1.

The results in Table 1 show that demethylation of bond X is more thermochemically favourable for both protonated and non-protonated forms of **4**. Furthermore, the difference in BDE values for non-protonated **4** are small, which can result in a mixture of demethylated products, in particular for the homolytic cleavage. Thus, it can be argued that protonation of the carbonyl oxygen is necessary for the selective demethylation to proceed. After protonation, the reaction is kinetically driven by KI to the desired product.

### 2.3. Biological Testing

The compounds were tested against the wild *C. albicans* strain and a reference strain ATCC18804 [39,40,41]. The agar-well diffusion method was used to evaluate the zones of inhibition (ZOI). This was followed by the determination of minimum inhibitory concentrations (MIC) and minimum fungicidal concentrations (MFC) for the active compounds. The results are shown in Table 2.

As can be seen in Table 2, the ZOIs show that compounds **1**, **2** and **3** are active against *C. albicans* and the reference strain, but no activity was observed for derivatives **4**, **5** and **6**. The antifungal drug clotrimazole was used as a reference, and it gave ~27% larger ZOI for the wild type strain and 16% for the reference strain 18804. All the compounds were tested at 2 mg/mL concentration. Considering that clotrimazole is an optimised drug in clinical use, it can be stated that compounds **1**–**3** are quite active. According to the MIC results, compound **2** is more active than **1** and **3,** and, finally, the MFC measurements revealed that coumarins **1** and **2** are fungicidal with 1.0 and 0.5 mg/mL, respectively. Interestingly, the isoflavone **3** is only fungistatic. This activity of the coumarins makes them promising lead compounds for further development for antifungal treatment. Furthermore, compounds **1**, **2** and **3** also showed activity against the *Sclerotium* fungal strain qualitatively confirming their efficacy (data not shown).

### 2.4. Molecular Modelling

It is established that microorganisms produce a unique class of sterols such as ergosterol necessary for their growth and survival [42]. The absence of these sterols in mammalian host cells makes these pathways an excellent target for therapeutic antifungal design [43,44]. In this light, some clinically employed antifungals such as the azoles have been designed to disrupt CYP51, a cytochrome P450 enzyme responsible for the synthesis of the sterols [28,45,46]. Coumarin antifungal derivatives designed in silico against the CYP51 enzyme have also showed promising preliminary results [47,48]. It can therefore be hypothesised that CYP51 is the putative target of the active compounds **1**–**3** from *M. thonningii*.

The coumarins and isoflavones were docked to the crystal structure of CYP51 (Protein data bank ID: 4ZE1) [49]. The co-crystallised posaconazole was removed and redocked. The root-mean-square deviation (RMSD) between the heavy atoms of the co-crystallised ligand and its docked counterparts was 1.33 Å and 1.51 Å using ChemScore (CS) and GoldScore (GS) respectively, verifying the prediction power of the method.

**Figure 3:**
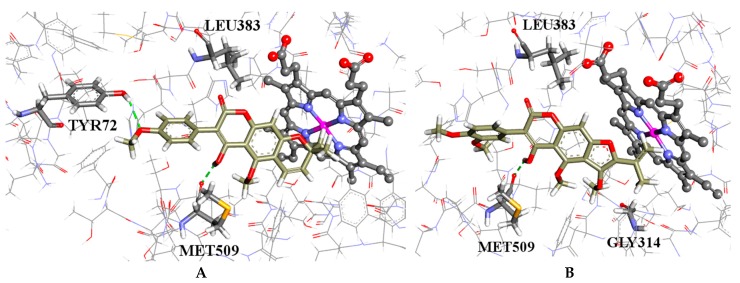
(**A**) the docked configuration of robustic acid (**1**) in the binding site of lanosterol 14α-demethylase (CYP51); and (**B**) the docked configuration of thonningine-C (**2**) in the binding site. Hydrogen bonds are shown as green dotted lines.

**Figure 4:**
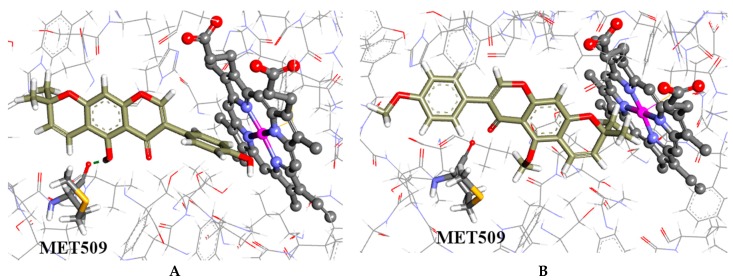
(**A**) the docked configuration of alpinumisoflavone (**3**) in the binding site of CYP51; and (**B**) the docked configuration of *O*,*O*-dimethylalpinumisoflavone (**4**) in the binding site. Hydrogen bonds are shown as green dotted lines.

It is predicted that the hydroxyl group of the coumarins bind to the backbone carboxylic moiety of methionine (MET509), which enables them to adopt a plausible binding configuration with methyl groups blocking the iron catalytic binding site as shown in Figure 3A,B. The isoflavones **3** and **5**, which have hydroxyl groups, also bind to the methionine but have their phenyl moieties inserted into the binding site; whereas the isoflavones **4** and **6** without the hydroxyl groups have similar configurations to the coumarins but do not form hydrogen bonds with the methionine, as shown in Figure 4A,B, respectively. This is a possible explanation why the coumarins are active but not the isoflavones.

Robustic acid (**1**) had the highest predicted affinity by CS and thonningine-C (**2**) for the GS results. Both of the coumarins gave better scores for both functions when compared to clotrimazole (see Table S2 in the Supplementary Materials). These predictions point toward CYP51 as the bio-molecular target of these compounds but with the very important caveat that experimental verification is needed. Nevertheless, the molecular modelling work supports a plausible hypothesis and points the way in which assays these compounds should be tested first.

### 2.5. Chemical Space

The calculated molecular descriptors molecular weight (MW), log *P*, hydrogen bond donors (HD), hydrogen bond acceptors (HA) polar surface area (PSA) and rotatable bonds (RB) given in Table S3 in the Supplementary Materials. All of the molecules are within the *drug-like* chemical space (for definition of *lead-like*, *drug-like* and Known Drug Space regions, see ref. [50] and Table S4). The molecular weight of the ligands is between 336.3 and 438.4 g·mol^−1^ and the log *P* values lie in the range of 3.6 and 4.6. These values demonstrate that the compounds are in a favourable area of chemical space for further development.

## 3. Materials and Methods

### 3.1. Isolation

#### 3.1.1. Plant Material

The seeds of *Milletia thonningii* were collected from the University of Ghana campus (Legon-Accra, Ghana). A voucher specimen was identified by Mr. D. K. Abbiw and then later Mr. J.Y. Amponsah at the Ghana National Herbarium, Department of Botany, University of Ghana. The voucher specimen (catalogue NO: 14832) was deposited at the herbarium. The seeds were shade dried for three weeks and then pulverised. All solvents were analytical grade. Thin-layer chromatography (TLC) was performed on aluminium foil slides pre-coated gel (thickness 0.2 mm, type Kiesegel 60 F_254_ Merck, Darmstadt, Germany) using petrol/ethyl acetate (10%, 15%, 20%, 25%, 30% and 35%); detection was performed using UV light and iodine vapour. Column chromatography was carried out on aluminium oxide (Fluka, Seelze, Germany).

#### 3.1.2. Extraction and Isolation

The pulverised plant material (4.5 kg) was extracted using cold percolation with petroleum ether (2 L) for five days at 27 °C. The extract was filtered and concentrated using rotary evaporator to give a brown solid (18 g) and stored in a refrigerator. In addition, 15 g of this solid were dissolved in chloroform, mixed with aluminium oxide and dried at 50 °C. This was placed on a column (50 mm × 1200 mm) pre-packed with aluminium oxide. The column was first eluted with petrol (500 mL), and then with a 5% increment of ethyl acetate in petrol, and 500 mL for each increment. At 45% of ethyl acetate in petrol, TLC profiling showed two dominant fractions F1a and F1b. These fractions were purified using column chromatography (CC) (20 mm × 600 mm) with aluminium oxide and an increasing amount ethyl acetate in petrol. Afterwards, the defatted plant material was also extracted with ethyl acetate (2 L) for a week at 27 °C. A yellow mixture (20 g) was obtained after solvent removal and also stored in a refrigerator. CC of 15 g of the yellow mixture was conducted as above. At 80% of ethyl acetate in petrol, TLC profiling showed four dominant fractions—F2a, F2b, F2c and F2d. These fractions were also purified on CC. The compounds were characterised by TLC, mass spectrometry, IR, ^1^H and ^13^C-NMR spectra and compared to published data. F1a and F2b were found to be the same compound and identified as isoflavone (**4**) (4.8 g). F1b and F2c were identified as isoflavones (**3**) (1.2 g) and (**5**) (23 mg), respectively. F2a and F2d were also identified as coumarin (**1**) (1.4 g) and (**2**) (642 mg), respectively.

*Robustic Acid* (**1**): Colourless block crystal, melting point (mp.) 207–209 °C. IR ν_max_: 3381, 1701, 1612 cm^−1^; Gas chromatography tandem Mass spectrometry (GC-MS) *m*/*z* 380, 365; ^1^H-NMR (400 MHz, CDCl_3_) δ: 9.91 (1H, s), 7.49 (2H, d, *J* = 8 Hz), 6.98 (2H, d, *J* = 8 Hz), 6.64 (1H, s), 6.53 (1H, d, *J* = 10 Hz), 5.80 (1H, d, *J* = 10 Hz), 3.98 (3H, s), 3.83 (3H, s), 1.49 (6H, s); ^13^C-NMR (100 MHz, CDCl_3_) δ: 162.2, 159.7, 158.6, 156.8, 153.5, 151.8, 131.1, 131.4, 123.0, 113.2, 114.7, 110.3, 103.6, 101.5, 101.4, 98.0, 64.0, 54.9, and 27.6. This is in agreement with published data [30,31].

*Thonningine-C* (**2**): Yellow granules; mp. 200–203 °C; IR ν_max_: 3288, 3093, 1716, 1658, 1620 cm^−1^; GC-MS *m*/*z* 437; ^1^H-NMR (400 MHz, CDCl_3_) δ: 10.23 (1H, s), 7.48 (1H, s), 7.45 (1H, d, *J* = 8 Hz), 7.00 (1H, s), 6.99 (1H, d, *J* = 8 Hz), 6.90 (1H, s), 6.89 (1H, m), 3.94 (1H, s), 3.91 (1H, s), 3.90 (1H, s), 3.85 (1H, s), 2.23 (1H, s); ^13^C-NMR (100 MHz, CDCl_3_) δ: 165.0, 160.1, 158.8, 158.5, 158.3, 143.8, 131.1, 122.1, 113.0, 110.4, 103.9, 102.1, 95.8, 64.7, 54.7, and 19.9. This is in agreement with published data [32].

*Alpinumisoflavone* (**3**): Yellow needle-like crystal; mp. 204–207 °C; IR ν_max_: 3454, 1654, 1614, and 1573 cm^−1^; GC-MS *m*/*z*: 336, 321; ^1^H-NMR (400 MHz, CDCl_3_) δ: 13.11 (1H, s), 7.82 (1H, s), 7.39 (2H, d, *J* = 8 Hz), 6.88 (2H, d, *J* = 8 Hz) 6.71 (1H, d, *J* = 10 Hz), 6.33 (1H, s), 5.64 (1H, d, *J* = 10 Hz), 5.25 (1H, s), 4.25 (1H, s), 3.93 (1H, s), 1.47 (6H, s); ^13^C-NMR (100 MHz, CDCl_3_) δ: 180.4, 159.0, 156.8, 156.3, 155.4, 152.0, 129.8, 127.6, 123.0, 122.5, 115.1, 114.9, 105.6, 105.1, 94.3, and 27.8. This is in agreement with published data [30,51].

*O,O-dimethylalpinumisoflavone* (**4**): Colourless granules (8.9 g); mp. 137–139 °C; IR ν_max_: 1636, 1605, 1512, 1389, 1362, 1244, 1067 cm^−1^, GC-MS *m*/*z*: 364, 349; ^1^H-NMR (400 MHz, CDCl_3_) δ: 7.76 (1H, s), 7.47 (2H, d, *J* = 8 Hz), 6.97 (2H, d, *J* = 8 Hz), 6.76 (1H, d, *J* = 10 Hz), 6.60 (1H, s), 5.74 (1H, d, *J* = 10 Hz), 3.83 (3H, s), 3.90 (3H, s),1.47 (6H, s); ^13^C-NMR (100 MHz, CDCl_3_) δ: 173.3, 157.8, 157.0, 156.3, 154.1, 148.6, 129.0, 128.6, 123.9, 122.6, 114.4, 112.2, 111.6, 111.5, 98.9, 61.0, 53.6, and 26.6. This is in agreement with published data [30,31].

### 3.2. Synthesis

#### 3.2.1. Synthesis of 4′-*O*-methylalpinumisoflavone (**5**)

To a solution of *O*,*O*-dimethylalpinumisoflavone (**4**) (1.00 g, 2.75 mmol) in 30 mL of ethyl acetate, KI (5.10 g, 30.73 mmol) was added, followed by formic acid (30 mL) as described by Mustafa et al. for the demethylation of khellin and visnagen [33]. This mixture was refluxed for 110 min. After the reflux, 50 mL of water was added and the product precipitated. The product was purified by chromatography (ethyl acetate/petroleum ether, 1:5) to yield 780 mg of 4′-*O*-methylalpinumisoflavone (**5**) as pale yellow needle-like crystals. mp.: 136–138 °C; IR ν_max_: 3423, 1651, 1614, 1512 cm^−1^; GC-MS *m*/*z*: 350, 335; ^1^H-NMR(400 MHz, CDCl_3_) δ: 13.16 (1H, s), 7.82 (1H, s), 7.47 (2H, d, *J* = 8 Hz), 7.00 (2H, d, *J* = 8 Hz), 6.75 (1H, d, *J* = 10 Hz), 6.34 (1H, s), 5.64 (1H, d, *J* = 10 Hz), 3.85 (3H, s), 1.48(6H, s); ^13^C-NMR (100 MHz, CDCl_3_) δ: 179.5, 158.4, 158.1, 155.9, 155.5, 151.0, 128.7, 126.7, 122.1, 121.6, 114.1, 112.7, 104.7, 104.2, 93.4, 53.9, and 26.9. This is in agreement with published data [30].

#### 3.2.2. Synthesis of Acetyl-4′-methylalpinumisoflavone (**6**)

A solution of 4′-*O*-methylalpinumisoflavone (**5**) (253 mg, 0.72 mmol) in 20 mL of acetic anhydride was refluxed for 120 min. After the reflux, a solution of sodium bicarbonate (10 g, 119 mmol) in 100 mL of water was added to the mixture to neutralise the acetic acid produced, which resulted in the precipitation of the product. The product was filtered and purified by chromatography (ethyl acetate/petroleum ether, 1:5) to afford 146 mg of acetyl-4′-methylalpinumisoflavone (**6**) as a colourless block crystal. mp.: 204–206 °C; IR ν_max_: 1766, 1635, 1608, 1514 cm^−1^; GC-MS *m*/*z*: 393, 335; ^1^H-NMR(400 MHz, CDCl_3_) δ: 7.75 (1H, s), 7.41 (2H, d, *J* = 8 Hz), 6.96 (2H, d, *J* = 8 Hz), 6.71 (1H, s), 6.51 (1H, d, *J* = 10 Hz), 5.78 (1H, d, *J* = 10 Hz), 2.45 (3H, s), 3.83 (3H, s),1.49 (6H, s); ^13^C-NMR (100 MHz, CDCl_3_) δ: 175.0, 169.3, 159.6, 158.1, 157.6, 150.8, 145.0, 132.0, 130.4, 125.7, 124.0, 115.2, 114.0, 113.0, 102.2, 94.0, 55.3, 28.4, and 21.1.

### 3.3. Bioactivity Studies

*C. albicans* were isolated and identified at the Komfo Anokye Teaching Hospital at Kumasi, Ghana. The organisms were brought to the Microbiology Department of the Centre for Plant Medicine Research (CPMR) at Mampong-Akuapem, Ghana.

#### 3.3.1. Preparation of Solutions and Media

20 mg of all the compounds were dissolved in DMSO (dimethylsulphoxide, 10 mL of 20%, (Sigma-Aldrich, D5879, Münich, Germany)), and 100 µg/mL solution of clotrimazole was also prepared using DMSO (20%). Nutrient agar, bacteriological peptone (Sigma-Aldrich, P0556) and Sabouraud 4% glucose agar (Fluka Biocheika 84088, Sigma-Aldrich, Bangalore, India) were prepared according to the manufacturer’s instructions. The stock cultures of *C. albicans* were subcultured onto fresh Nutrient agar plates and then incubated for 24 hours and stored in a refrigerator overnight.

#### 3.3.2. Determination of the Potency of the Compounds

The agar-well diffusion method was used to investigate the antimicrobial properties of the isoflavones and the coumarins [52,53].

About three to five colonies of the same morphological type of the microbes were suspended in test tubes containing 5 mL of sterilised bacteriological peptone and incubated at 37 °C for 16 h. After the 16 hours, the microorganisms were subcultured in sterilised bacteriological peptone water and incubated again for another two hours. The turbidity was adjusted to a 0.5 McFarland standard [52]. The dried Sabouraud 4% glucose agar plates were then flooded with the fungi and the surface was allowed to dry. A sterilised cock borer of an internal diameter of 6 mm was used to punch wells in the medium. The compounds were then dispensed into their respective labelled wells with concentrations of 2 mg/mL for all the compounds tested; and DMSO (20%) was dispensed into its labelled well as a negative control whilst clotrimazole (2 mg/mL) was dispensed as a positive control. Triplicates of each plate with the same labelled compounds were made for each fungus. The plates were kept in the refrigerator overnight for complete diffusion. The plates were then incubated at 35 °C for 48 h. After the incubation period, the diameter of each inhibition zone was measured in millimeters (mm) with a metre rule.

#### 3.3.3. Determination of Minimum Inhibitory Concentration and the Fungicidal or Fungistatic Effect of the Active Compounds

The MICs of the active compounds were determined using the microplate dilution method as described by Eloff [54]. A hundred microliters (100 µL) of 2 mg/mL solution of an active compound was placed in the first well of the second row of the 96-well microplate. About 100 µL of the same active compound was added to 100 µL of sterilised bacteriological peptone water in the next well of the second column. This was mixed with a micropipette and 100 µL of this dilution was added to the well in the next column, which contained 100 µL of sterilised bacteriological peptone water. This process was repeated to produce two-fold serial dilutions in the original concentration. About 100 μL of DMSO (20%) was placed in the last column. This process was repeated for three rows on the 96-well microplate. In addition, 100 µL of clotrimazole was placed in the last row. Furthermore, 100 μL of subcultured fungi were placed in all the wells except the first row, which contains only peptone water. The plates were then covered and incubated at 35 °C for 48 h, after which 40 µL of iodonitrotetrazolium chloride (INT, 0.2 mg/mL) was added to each well and examined after 120 min of incubation. Fungi growth was indicated by a red colour of formazan, which is the reduced form of the INT. The minimum concentration at which there was no colour was taken as the minimum inhibitory concentration.

Samples from the wells with no colour, and, therefore, wells with no observable fungi growth were incubated at 35 °C for 48 h on fresh Sabouraud 4% glucose agar plates using inoculation pins. The minimum concentration at which no growth of microbes was seen was taken as the minimum fungicidal concentration.

### 3.4. Thermochemical Calculations

The calculations were done using Gaussian 09 software suite [55] (Gaussian Inc., Wallingford, CT, USA) employing a restricted non-local B3LYP functional hybrid method [56,57,58]. The standard 6-31+G (d, p) [59,60] basis set was used for the geometry optimisation and frequency analysis while the larger 6-311+G (2df, p) basis set was employed to perform the energy calculations. The zero-point vibrational energies (ZPE) were scaled using Wong’s value of 0.9804 [61]. In all cases, the normal modes revealed no imaginary frequencies, which indicate that they represent the minima on the potential energy surface. The PAs and BDEs were calculated as described by Foresman and Frisch [62].

### 3.5. Molecular Modelling

Compounds **1**–**6** were docked into the crystal structure of CYP51 (Protein Data Bank ID: 4ZE1 resolution 2.05 Å) [49], which was obtained from Protein Data Bank (PDB) [63,64]. The Scigress version FJ 2.6 program [65] (Fijitsu Limited, Kanagawa, Japan**)** was used to prepare the crystal structure for docking, hydrogen atoms were added, the co-crystallised ligand as well as crystallographic water molecules were removed. The Scigress software suite was also used to build the inhibitors and the MM2 [66] force field was used for structural optimisation. The centre of the binding was defined as the position the carbonyl oxygen of methionine 509 (*x* = 4.024, *y* = 15.088, *z* = 99.736) with a 10 Å radius. Hundred docking runs were allowed for each ligand with default search efficiency (100%). The GoldScore [67], and ChemScore [68,69], scoring functions were implemented using the parameter file for cytochromes P450 and heme-containing proteins [70] developed to predict binding modes and relative binding energies of the ligands using the GOLD v5.4 software suite (Cambridge Crystallographic Data Centre, Cambridge, UK). This heme-containing proteins parameter file was developed using data from the Cambridge Structural Database (CSD) and Protein Data Bank. The root square mean deviation (RSMD) of the co-crystallised ligand for CSD-ChemScore, CSD-GoldScore, PDB-ChemScore, and PDB-GoldScore were 1.333 Å, 1.710 Å, 1.595 Å and 1.509 Å, respectively. The CSD-Chemscore, which had the best RSMD value, was used for further analysis. The docking scores of the compounds are given in Table S1 in the supplementary materials.

The QikProp 3.2 [71] software package (Schrödinger LLC, New York, NY, USA) was used to calculate the molecular descriptors of the molecules. The reliability has been established for the calculated descriptors [72].

## 4. Conclusions

Previous studies have focused on azoles, polyenes, griseofulvin, flucytosine and allylamine molecular scaffolds for antifungal design, e.g., the clinically used drugs voriconazole, ketoconazole and fluconazole are derived from azole containing molecules and terbinafine from allylamine [10]. However, not much attention has been paid to the development of coumarins as antifungals. This study demonstrates their potential for antifungal drug development.

Robustic acid (**1**) and thonnigine-C (**2**) were active against the fungal strains tested. The predicted mechanism of action showed that the hydroxyl group binds to the methionine backbone of CYP51 helping the coumarins to adopt a pose that blocks access to the catalytic site of the enzyme. Interestingly, the coumarins under study appear to be non-toxic in mammalian systems due to the numerous traditional medicinal use of the *M. thonningii*. This non-toxic nature presents these coumarins as good antifungal leads, which might be developed further as antifungal drugs and food preservatives. This study extends the number of coumarins with antifungal properties reported [23,24,25,26] in addition to providing direction for rational design of coumarins as antifungal agents.

## Figures and Tables

**Table 1:** Bond dissociation energies (BDEs) in kcal/mol.

Bond Dissociation	X	Y	Difference
Homolytic	51.2	54.0	2.8
Heterolytic	216.0	227.8	11.8
Homolytic-protonated	42.5	62.7	20.2
Heterolytic-protonated	111.4	167.6	56.2

**Table 2:** Zones of inhibition (ZOI), minimum inhibitory concentrations (MIC) and minimum fungicidal concentrations (MFC).

Compound	ZOI (mm)		MIC (mg/mL)		MFC (mg/mL)	
	WILD	18804	WILD	18804	WILD	18804
Robustic acid (**1**)	10.3	14.7	0.25	1.00	1.0	1.0
Thonningine-C (**2**)	10.3	14.7	0.13	0.25	0.5	0.5
Alpinumisoflavone (**3**)	10.7	15.0	0.25	0.50	F	F
*O*,*O*-dimethylalpinumisoflavone (**4**)	0	0	X	X	X	X
4-*O*-methylalpinumisoflavone (**5**)	0	0	X	X	X	X
Acetyl-4-*O*-methylalpinumisoflavone (**6**)	0	0	X	X	X	X
Clotrimazole	14.4	17.6	X	X	X	X
DMSO (20%)	0	0	X	X	X	X

Zones of inhibition = ZOI; minimum inhibitory concentrations = MIC; minimum fungicidal concentrations = MFC; WILD = wild *C. albicans* strain; 18804 = reference ATCC18804 strain; X = No data; F = Fungistatic.

## Supplementary Materials

Supplementary materials can be accessed at: http://www.mdpi.com/1420-3049/21/10/1369/s1. Table S1: Bond dissociation energies, Table S2: Results of the scoring function for the ligands, Table S3: The calculated molecular descriptors for the ligands, Table S4: Definition of lead-like, drug-like and known drug space (KDS) in terms of molecular descriptors.
