# Supplementary material for: Coumarin Antifungal Lead Compounds from Millettia thonningii and Their Predicted Mechanism of Action
Source: Molecules. 2016 Oct 15;21(10):1369. doi: 10.3390/molecules21101369 (PMC6274499; doi:10.3390/molecules21101369)
Supplement: Supplementary file 1 [file molecules-21-01369-s001.pdf]

Coumarin Antifungal Lead Compounds from *Millettia thonningii* and Their Predicted Mechanism of Action

Daniel M. Ayine-Tora, Robert Kingsford-Adaboh, William A. Asomaning, Jerry J.E.K. Harrison, F.C Mills-Robertson, Yahaya Bukari, Patrick O. Sakyi, Sylvester Kaminta and Jóhannes Reynisson

Table S1. Bond Dissociation energies.

Molecule	Energy	ZPE	BDE (kcal/mol)	Cal. PA (kcal/mol)
Homolytic cleavage				
Isoflavone 4	−1226.859664	0.372745		
X	−1186.910352	0.331539	51.21990931	
Y	−1186.906032	0.331611	53.97504769	
CH_3	−39.85652694	0.029821		
Heterolyic cleavage				
Isoflavone 4	−1226.859664	0.372745		
X	−1187.012496	0.33063	216.0468571	
Y	−1186.993448	0.330344	227.8236545	
CH_3	−39.49236094	0.03139		
Protonation on carbonyl oxygen				
Homolytic cleavage				
Isoflavone 4	−1227.253371	0.386664		238.493
X	−1187.317698	0.345246	42.53022312	
Y	−1187.285263	0.344971	62.71427052	
CH_3	−39.85652694	0.029821		
Heterolyic cleavage				
Isoflavone 4	−1227.253371	0.386664		
X	−1187.574148	0.345679		
Y	−1187.482986	0.344166		
CH_3	−39.49236094	0.03139		
Protonation on methoxy oxygen				
X	−1227.253372	0.386663		238.493 *
Y	−1227.178908	0.384684		192.984

* When the methoxy oxygen on bond X was protonated, the proton moved to the carbonyl oxygen after optimization.

Table S2. Results of the scoring function for the ligands.

Compound	CSD		PDB		Activity
	CS	GS	CS	GS	
Co-crystallised ligand (Posaconazole)$^{\alpha}$	46.9	112.2	53.8	116.3	
Clotrimazole	27.6	60.8	26.4	53.5	
Robustic acid (**1**)	43.2	61.4	43.8	61.6	Fungicidal
Thonningine-C (**2**)	39.4	68.3	39.3	67.2	Fungicidal
Alpinumisoflavone (**3**)	39.7	56.9	39.9	54.6	Fungistatic
O,*O*-dimethylalpinumisoflavone(**4**)	41.0	58.4	40.8	59.7	Inactive
4-*O*-methylalpinumisoflavone (**5**)	42.3	61.6	42.4	61.2	Inactive
Acetyl-4-*O*-methylalpinumisoflavone(**6**)	40.5	62.6	39.4	62.5	Inactive

Table S3. The calculated molecular descriptors for the ligands.

Ligand	MW	HB Donor	HB Acceptor	Log *P*	PSA	Rot. Bonds
robustic acid (**1**)	380.4	1	5.5	4.0	73.9	4
thonningine-C (**2**)	438.4	1	6.8	4.3	84.7	7
alpinumisoflavone (**3**)	336.3	1	3.8	3.6	78.2	3
O,*O*-dimethylalpinumisoflavone (**4**)	364.4	0	4.8	4.6	46.7	3
4-*O*-methylalpinumisoflavone (**5**)	350.4	0	3.8	4.4	63.9	3
acetyl-4-*O*-methylalpinumisoflavone (**6**)	392.4	0	6.5	3.8	71.8	3

Table S4. Definition of lead-like, drug-like and Known drug space (KDS) in terms of molecular descriptors. The values given are the maxima for each descriptor for the volumes of chemical space used.

	Lead-Like Space	Drug-like Space	Known Drug Space
Molecular weight ($g \cdot mol^{-1}$)	300	500	800
Lipophilicity (Log *P*)	3	5	6.5
Hydrogen bond donors (HD)	3	5	7
Hydrogen bond acceptors (HA)	3	10	15
Polar surface area ($Å^2$) (PSA)	60	140	180
Rotatable bonds (RB)	3	10	17
